# Miscellany

**Published:** 1896-04

**Authors:** 


### Miscellany.

THE Mellier Drug Company, of St. Louis, has issued a little brochure entitled Mellier's Galaxy of Eminent Medical Men, copies of which will be posted to any physician who will mention this journal in his request. A specimen of these portraits may be found on advertising page xiv. of this issue of the JOURNAL. The address of Mellier Drug Company is 2112 Lucas place, St. Louis.

ACKNOWLEDGMENT.—The University Medical College of Kansas City, Mo., held its annual commencement Thursday, March 19, 1896. The courtesy of an invitation to attend the ceremonies is gratefully acknowledged.

THE Charles Roome Parmele Company has removed from 98 William street to 36 Platt street, New York. The object of this change is to obtain more commodious accommodations for the growing business of this enterprising house.

CHARLES WOOD FASSETT, secretary of the American Medical Publishers' Association, has just issued a revised edition of the Medical Journal Exchange List, containing the names and addresses of all publications in the United States and Canada, devoted to medicine, surgery, pharmacy, hygiene, microscopy, and allied sciences. This list is printed upon adhesive paper, and is used extensively by publishers in mailing their exchanges, as well as by scientific writers in sending out reprints, etc. Price, $1.25 per dozen complete sheets. (Furnished free to members of the association.)

THE American Medical Association meets in Atlanta, Ga., May 5, 1896, and all delegates to this society should embrace the opportunity of visiting "Lookout Inn," a magnificent and historic spot on the crest of Lookout Mountain, overlooking Chattanooga, Tenn. A special invitation is extended to physicians to spend a week with us, en route to Atlanta, and a complimentary rate will be made for the occasion. Trains up the mountain make close connections in both the Central and Union Depots, Chattanooga, running through to the Inn without change. For further information and handsome, illustrated booklet, address M. S. Gibson, Lookout Mountain, Tennessee.

---

THE DR. L. CH. BOISLINIÉRE PRIZE ESSAY FUND.—At a general meeting of the medical profession of St. Louis, held on January 13, 1896, the undersigned were appointed a committee to determine upon and institute some suitable memorial in honor of our lamented and revered colleague, Dr. L. Ch. Boisliniére, and to solicit subscriptions from physicians and the public at large, in order to properly accomplish this object.

After mature deliberation and free discussion, both in and out of the committee, as to the shape this memorial should take, it has been determined to found a Boisliniére prize essay, on some subject connected with obstetrics and gynecology, the award to be made triennially and to be open to the competition of regular physicians residing in the United States.

The committee believes that a memorial of this nature will not only keep in perpetual remembrance the name of our honored friend, but also, in thus fostering medical science and encouraging talent and industry, we shall be working in his spirit and furthering objects that were always very near to his own heart.

The committee has also been very fortunate in obtaining the consent of the St. Louis Obstetrical and Gynecological Society to act as trustee for such sums as we may be able to secure, and this society will also undertake the administration of the fund under the conditions set forth above.

Mr. W. H. Lee, president of the Merchants Laclede National Bank has kindly agreed to assume the duties of honorary treasurer, and will duly acknowledge subscriptions. All checks should be made payable to Mr. Lee.

Committee: W. A. Hardaway, M. D., chairman; E. H. Gregory, M. D., J. K. Bauduy, M. D., Jno. P. Bryson, M. D., Walter B. Dorsett, M. D., secretary; E. S. Smith, M. D., J. Friedman, M. D.

